# A multi-epitope fusion antigen candidate vaccine for Enterotoxigenic *Escherichia coli* is protective against strain B7A colonization in a rabbit model

**DOI:** 10.1371/journal.pntd.0010177

**Published:** 2022-02-09

**Authors:** Richard M. Jones, Hyesuk Seo, Weiping Zhang, David A. Sack

**Affiliations:** 1 Johns Hopkins Bloomberg School of Public Health, Department of International Health, Baltimore, Maryland, United States of America; 2 University of Washington, Department of Microbiology, Seattle, Washington, United States of America; 3 University of Illinois at Urbana-Champaign, Department of Pathobiology, Urbana, Illinois, United States of America; University of Texas Medical Branch, UNITED STATES

## Abstract

Enterotoxigenic *Escherichia coli* (ETEC) strains are a leading cause of children’s and travelers’ diarrhea. Developing effective vaccines against this heterologous group has proven difficult due to the varied nature of toxins and adhesins that determine their pathology. A multivalent candidate vaccine was developed using a multi-epitope fusion antigen (MEFA) vaccinology platform and shown to effectively elicit broad protective antibody responses in mice and pigs. However, direct protection against ETEC colonization of the small intestine was not measured in these systems. Colonization of ETEC strains is known to be a determining factor in disease outcomes and is adhesin-dependent. In this study, we developed a non-surgical rabbit colonization model to study immune protection against ETEC colonization in rabbits. We tested the ability for the MEFA-based vaccine adhesin antigen, in combination with dmLT adjuvant, to induce broad immune responses and to protect from ETEC colonization of the rabbit small intestine. Our results indicate that the candidate vaccine MEFA antigen elicits antibodies in rabbits that react to seven adhesins included in its construction and protects against colonization of a challenge strain that consistently colonized naïve rabbits.

## Author summary

Diarrheal diseases remain a major health threat around the world. Enterotoxigenic *E*. *coli* (ETEC) are a group of bacteria that are among the leading causes of diarrheal disease in travelers and children under five. One characteristic of ETEC are the diverse surface proteins used to colonize the gut of mammals. We have developed a single protein vaccine candidate called a multi-epitope fusion antigen (MEFA) that elicits antibody production in mice and pigs against seven diverse colonization factors. Here, we took advantage of a rabbit model of ETEC colonization to determine if immunization with this MEFA can provide direct protection against colonization of the small intestine. Our results indicate that immunization of rabbits with the candidate vaccine elicits antibodies that bind to seven colonization factors included in its construction and protects against colonization of a challenge strain that consistently colonized unvaccinated rabbits. This study provides additional evidence to support the MEFA strategy for generating a broadly protective vaccine for ETEC and other diverse enteric pathogens.

## Introduction

Enterotoxigenic *Escherichia coli* (ETEC) is a leading cause of children’s and travelers’ diarrhea across the globe [1]. More pressing, these strains are a leading cause of death in children under 5 years old in developing country settings [2–4]. ETEC strains are characterized by the presence of enterotoxins and adhesins. The enterotoxins are defined as heat-stable (ST) and/or heat-labile toxin (LT) [5]. The delivery of either toxin in the host small intestine leads to interference with cell signaling and release of water and electrolytes from the epithelium as with cholera toxin. In addition to the expression of these toxins, ETEC strains utilize a large number of immunologically heterologous adhesins known as colonization factor antigens (CFA) [5]. Attachment and adherence by ETEC adhesins to host receptors allow for colonization of the organism in the host small intestine, eventually leading to establishment of infection.

In the era of next-generation sequencing, genetic approaches have been employed to identify the most important and widespread CFAs [6, 7]. These studies inform the global landscape of ETEC infection, including which strains are dominant and details about the genetic basis of this pathovar’s diversity. In a large multi-site study, certain ETEC pathovars were commonly linked to more severe forms of diarrhea in children under five years old [4]. Further investigation of the CFA profile of these strains showed that major colonization factors (Colonization Factor Antigen I [CFA/I] or Coli Surface [CS] antigens CS1-CS6) were detected in 66% of LT/ST and ST-only cases of moderate to severe diarrhea [8]. The data from such analyses suggest that one strategy for prevention of ETEC infection is a vaccine that elicits an immune response against multiple prevalently expressed CFAs.

As ETEC infections are a global problem for children and travelers, there has been a long-term effort to develop a licensed ETEC vaccine [9, 10]. However, the heterologous nature of these organisms presents inherent difficulties for vaccine development. Investigators have utilized strategies such as combining multiple strains overexpressing CFs, which have been shown to be safe, reactive, and immunogenic in human trials [11–15]. Similarly, researchers have developed a combined *Shigella*/ETEC vaccine by using attenuated *Shigella* strains expressing ETEC CFs from the chromosome [16]. These approaches show promise and are undergoing various levels of clinical trials. However, one hurdle that remains is a lack of immunogenicity of these candidate oral vaccines in children living in developing countries [17, 18] as well as the lack of an antigen inducing antibodies to neutralize ST toxin [14].

Adopting an epitope- and structure-based multi-epitope fusion antigen (MEFA) vaccinology platform, a polyvalent vaccine adhesin antigen was generated using epitopes from characteristic colonization factor antigens (CFA) associated with ETEC infection [19]. Briefly, assisted with computation biology and protein modeling, epitopes from CS1-6 were identified and fused onto the CFA/I subunit CfaB backbone to generate a single protein antigen [19]. Previous work has shown that this fusion antigen (CFA/I/II/IV MEFA) alone or combined with administration of a separate toxoid fusion protein which included three copies of ST toxoid STa_N12S_ and a monomeric LT mutant (one A subunit mutated at the 192 and 211 sites fused to one B subunit in a single peptide) induced broadly neutralizing antibodies in mice and protected against ETEC diarrhea caused by strains that produce STa or LT toxins [19–21]. However, protection against ETEC colonization of small intestines was not quantified in these studies but is known to be a crucial step for the establishment of disease in humans [22]. Since earlier studies have investigated the colonization of *Shigellae*, *Vibrio cholerae* and *E*. *coli* in a rabbit small intestine model, we adapted a non-surgical rabbit colonization model to study immune protection against ETEC colonization in rabbits [23–25]. The rabbit model has previously been utilized to investigate enteric colonization of *V*. *cholerae*, including the role of toxin, mechanisms of protection, and efficacy of antibiotic treatment [26–28]. Using this model of enteric colonization, we tested the ability for MEFA-based vaccine protein CFA/I/II/IV, in combination with dmLT adjuvant, to induce broad immune responses and to protect from ETEC colonization of the rabbit small intestine.

## Materials and methods

### Ethics statement

All rabbit studies complied with the 1996 National Research Council guidelines and USDA Animal Welfare Act and Regulation and were approved and supervised by Johns Hopkins University Institutional Animal Care and Use committee under approved protocol number RB16H449.

### Bacterial strains

Strains are listed with their descriptions in Table 1. The rabbit challenge strains were obtained from PATH from cGMP production cell banks, produced at WRAIR and have been used in a number of human challenge models [29, 30].

**Table 1:** *Escherichia coli* strains.

Strains	Relevant properties	Sources
H10407	O78:H11; CFA/I, LT, STa	PATH
E24377A	O139:H28; CS1/3, LT, STa	PATH
B7A	O148:H28; CS6, LT, STa	PATH
THK38/pEU405	CS1	Emory University [31]
DH5α/pEU588	CS2	Emory University [32]
E116 (E19446)	CS3, LT, STa	Univ. of Gothenburg
E106 (E11881/9)	CS4/CS6, LT, STa	Univ. of Gothenburg
UM 75688	CS5/CS6, LT, STa	Johns Hopkins Univ.
2423 ETP98066	CS6, LT, STa	Washington Univ. [33]
9558	CS6 CssA subunit protein	This study
9471	toxoid fusion 3xSTa_N12S_-mnLT_R192G/L211A_ protein	Univ. of Illinois [34]
9472	CFA/I/II/IV MEFA protein	Univ. of Illinois [19]

*E*. *coli* strains used in rabbit colonization model, antibody adherence inhibition assays, extraction of adhesins or expression of CS6 CssA recombinant protein as ELISA coating antigens for antibody titration, and expression of recombinant proteins as rabbit vaccination antigens in the study.

### Rabbit intradermal (ID) or intramuscular (IM) immunization

Groups of naïve New Zealand White rabbits (Covance Research Products, Inc., Denver, PA) were immunized with three doses of CFA/I/II/IV MEFA protein (100 μg), along with dmLT adjuvant (1μg). Animals were injected either intradermally or intramuscularly. Serum was collected using Covidien Corvac serum separation tubes (Medtronic, Minneapolis, MN) spun for 10 minutes at 3,000 x *g* and titers against each of the CFAs and LT toxin (with CT used as coating antigen, details below) determined by ELISA.

### Rabbit serum anti-adhesin and antitoxin IgG antibody titration

Anti-CFA/I, -CS1, -CS2, -CS3, -CS4, -CS5, -CS6 anti-adhesin and anti-LT antitoxin IgG antibody titers were measured in ELISAs as previously described [21]. Briefly, wells of 2HB plates (Thermo Scientific, Rochester, NY) were coated with 100 ng of CFA/I, CS1, CS2, CS3, CS4, or CS5 adhesin heat-extracted from ETEC field isolates or recombinant *E*. *coli* strains (Table 1), CS6 subunit CssA recombinant protein, or cholera toxin (CT; Sigma; CT is commonly used as the ELISA coating antigen for anti-LT antibodies titration due to the high homology [35]). After blocking with 10% nonfat dry milk for 1 hr, plates were incubated with diluted rabbit serum (two-fold dilution from 1:400) at 37°C for 1h followed by washing with PBS containing 0.05% (v/v) Tween 20. Horseradish peroxidase (HRP) conjugated goat anti-rabbit IgG antibodies (1:5,000; Sigma) as secondary antibody, and 3,3’,5,5’-tetramethylbenzidine (TMB) Microwell Peroxidase Substrate System (KPL, Gaithersburg, MD) were used to measure optical density (OD650). IgG titers were determined by the highest IgG sample dilution producing an OD reading above 0.3 after subtraction of background readings and were presented in log10 as previously described [21, 36]. Fold change (log_10_) was similarly reported for IM and ID injections.

### Rabbit IgG antibody bacterial adherence inhibition assay

Rabbit serum samples were examined for *in vitro* antibody inhibition activity against ETEC adherence to Caco-2 cells (ATCC, #HTB-37TM) as described previously [21]. Briefly, 1 × 10^6^ CFU ETEC bacteria expressing CS6, or B7A at the MOI of 10 bacteria per cell were pre-treated with 4% mannose and mixed with 15 μl of rabbit serum samples. After shaking for 30 min at 50 rpm, each serum-and-bacteria mixture was brought to 300 μl with Eagle’s Minimum Essential Medium (EMEM, ATCC, catalog number: 30–2003™) and added to 1 × 10^5^ cells of Caco-2 cells in 48-well plates. The plates were incubated in a 5% CO_2_ incubator at 37°C for 1 h and gently rinsed with PBS to remove non-adherent bacteria. Caco-2 cells were dislodged with 0.5% Triton X-100 and *E*. *coli* bacteria adherent to Caco-2 cells were collected. Dislodged cells with adherent bacteria were serially diluted and plated on LB agar plates. After overnight growth at 37°C, bacteria CFUs were counted.

### Rabbit immunization and challenge studies

Naïve, previously challenged, or vaccinated rabbits were used for the colonization/protection challenge study. Previously challenged rabbits were used to generate immunogenic comparator groups—naïve rabbits were challenged with ETEC bacteria and allowed 2 weeks to return to baseline before re-challenge. Vaccinated rabbits received three doses with a 10-day interval. One dose consisted of 100 μg of CFA/I/II/IV MEFA protein and 1 μg of dmLT as adjuvant. After a 24 hour fast, rabbits were challenged or re-challenged with 10^9^, 10^10^, or 10^11^ colony-forming units of ETEC using oro-gastric gavage. The dose used for re-challenge was always 10^11^ CFU. Challenge gavage was preceded by administration of 15 ml of 5% sodium bicarbonate buffer and 0.5 mg/kg famotidine, followed by administration of the bacterial suspension in 10 ml of 1.5% sodium bicarbonate buffer and a final flush with an additional 5 ml of 1.5% sodium bicarbonate buffer. After 24 h the animals were sacrificed, and 10 cm intestinal sections were taken from the proximal and/or distal small intestine. These were opened, rinsed to remove feces and the contents of the intestinal lumen, vortexed in sterile PBS, and dilutions were plated on MacConkey agar (BD Difco, Franklin Lakes, NJ) for colony-forming units. After overnight incubation, a subset of the colonies (25% or 50 colonies) were confirmed to be the expected serotype via agglutination with the corresponding antiserum as described previously or were confirmed using colony PCR for toxin genes [15].

## Results

### Rabbits immunized intradermally or intramuscularly with CFA/I/II/IV MEFA protein with dmLT adjuvant developed IgG antibody responses to seven ETEC adhesins and LT toxin

**Fig 1:**
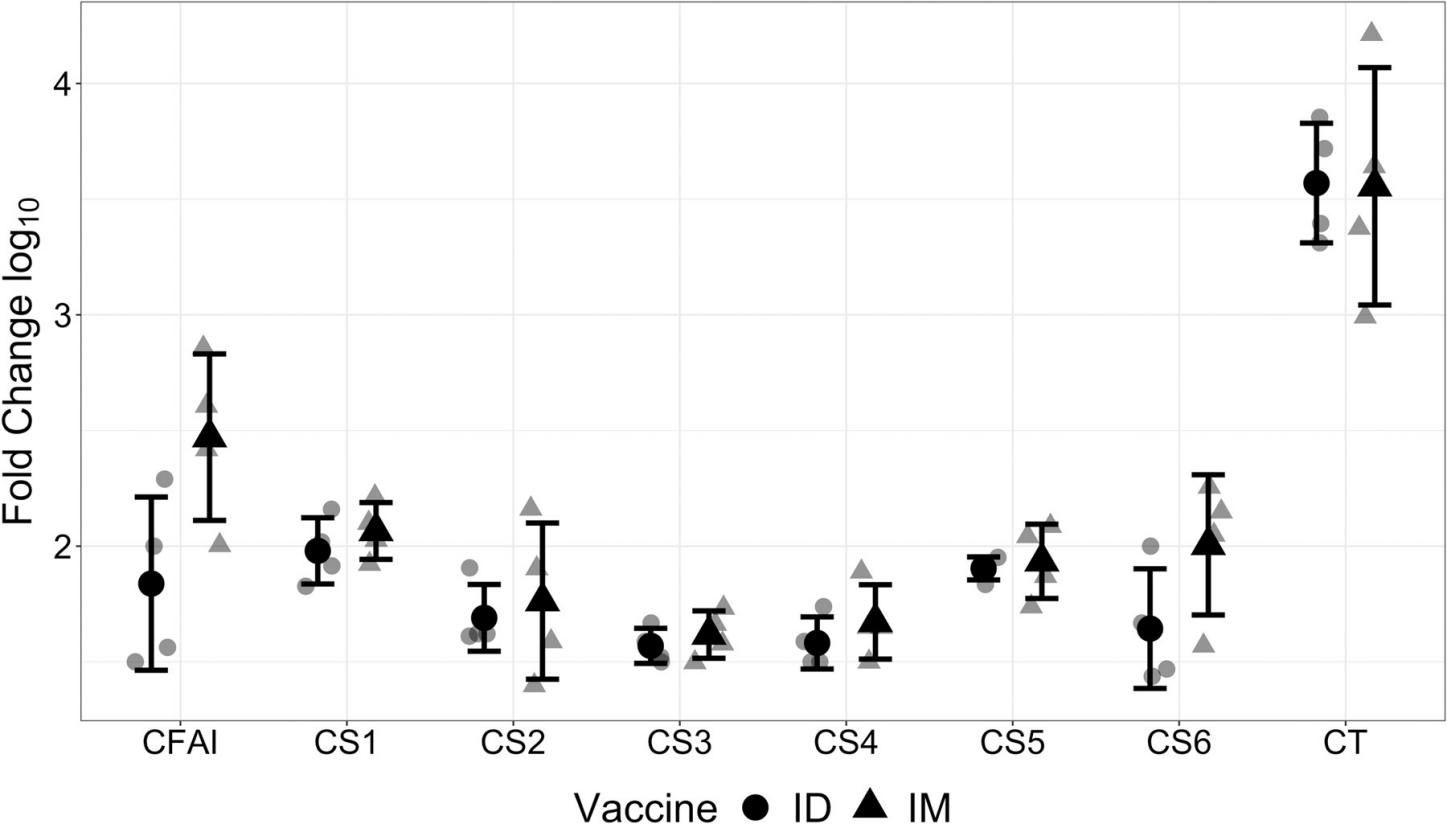
Fold change in IgG antibody titers when compared to control serum (from non-vaccinated rabbits) for various CFAs in rabbit serum. Serum samples of each rabbit intramuscularly (triangles) or intradermally (circles) vaccinated with CFA/I/II/IV MEFA protein, with dmLT adjuvant, were titrated in ELISAs with CFA/I, CS1, CS2, CS3, CS4, CS5 adhesin, CS6 CssA recombinant protein, or CT (for LTB) as coating antigens. Rabbits were immunized twice, with a period of two weeks between doses. Mean and standard deviations are shown in black, individual fold changes in gray.

Administration of the CFA/I/II/IV MEFA resulted in the production of antibodies against CFA/I, CS1, CS2, CS3, CS4, CS5, and CS6, the CFAs included in the fusion antigen as measured by ELISA. The CFA/I/II/IV MEFA protein was administered as described above both intradermally and intramuscularly with dmLT as an adjuvant. Antibody titers rose in response to both methods of administration and were not significantly different from one another (**Fig 1**). High antitoxin (to CT, which has high homology to LTB and is commonly used as coating antigen) titer rises were also seen in the rabbits immunized with CFA/I/II/IV MEFA, which was given with dmLT adjuvant. No anti-adhesin or antitoxin antibody responses were detected from the control rabbits.

### Naïve rabbits show primarily distal colonization of the small intestine by ETEC, with strain-to-strain variability

**Fig 2:**
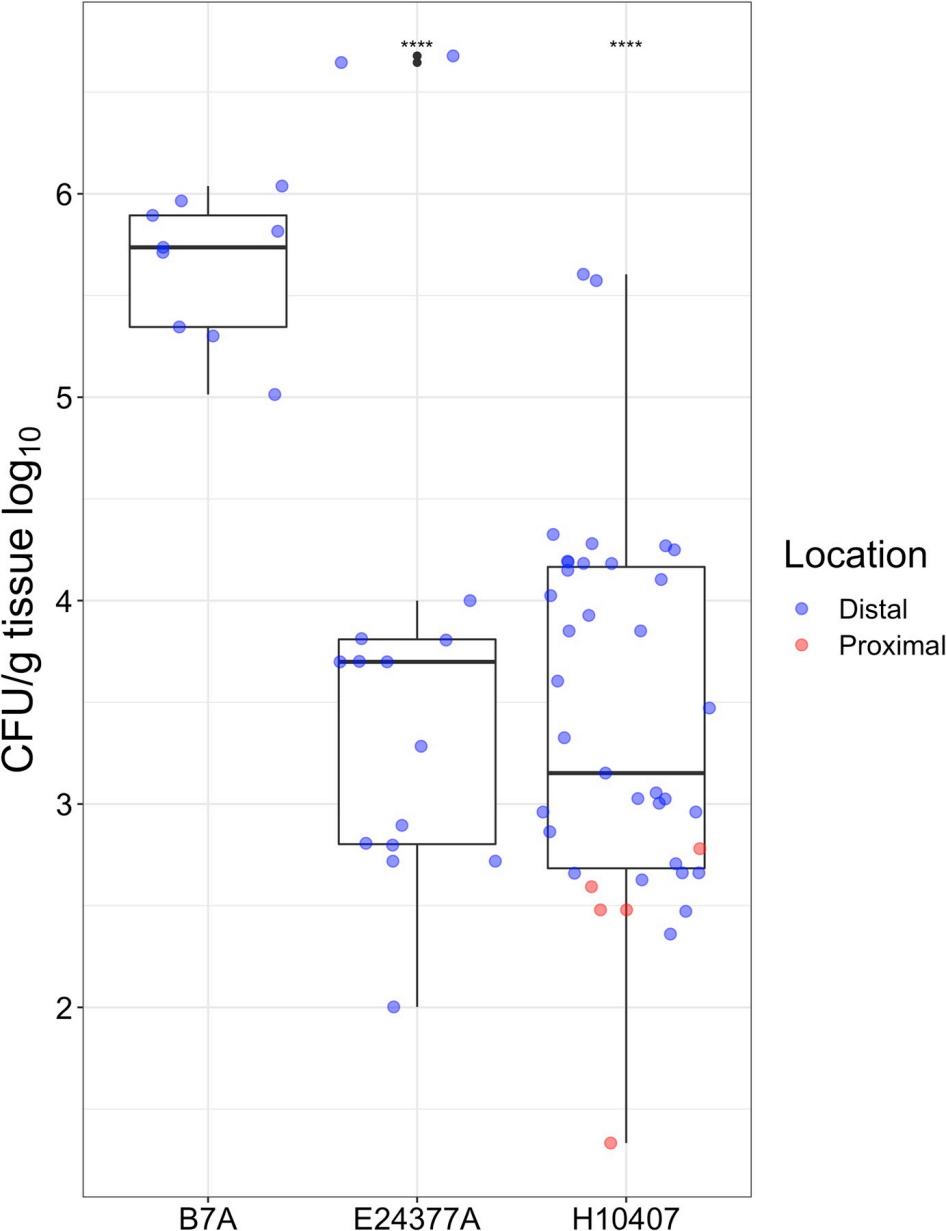
Colonization of naïve rabbits with ETEC strains, showing location of the small intestine sampled (distal: blue, proximal: red). Shown are plate counts for each rabbit (single points), with boxplots overlayed displaying the median and interquartile range in black. The strain of ETEC used for the challenge is listed at the bottom. ****: p < = 0.0001 when Student’s T-test performed with B7A.

After administration of ETEC to naïve rabbits via the bacterial challenge procedure described above, proximal and distal sections of the intestine were plated to determine ETEC colonization. For the strains tested (H10407, E24377A, B7A –see **Table 1)**, colonization was almost entirely distal, as the CFU/g from the proximal small intestine was negligible after plating on MacConkey. Naïve rabbits that were challenged with strains H10407 or E24377A showed high variability in CFU/g intestine within and between animals **(Fig 2)**. However, strain B7A showed a consistently high level of colonization in naïve animals.

### Rabbits previously challenged with B7A and those immunized with the CFA/I/II/IV MEFA protein have a significant decrease in B7A colonization compared to naïve animals

**Fig 3:**
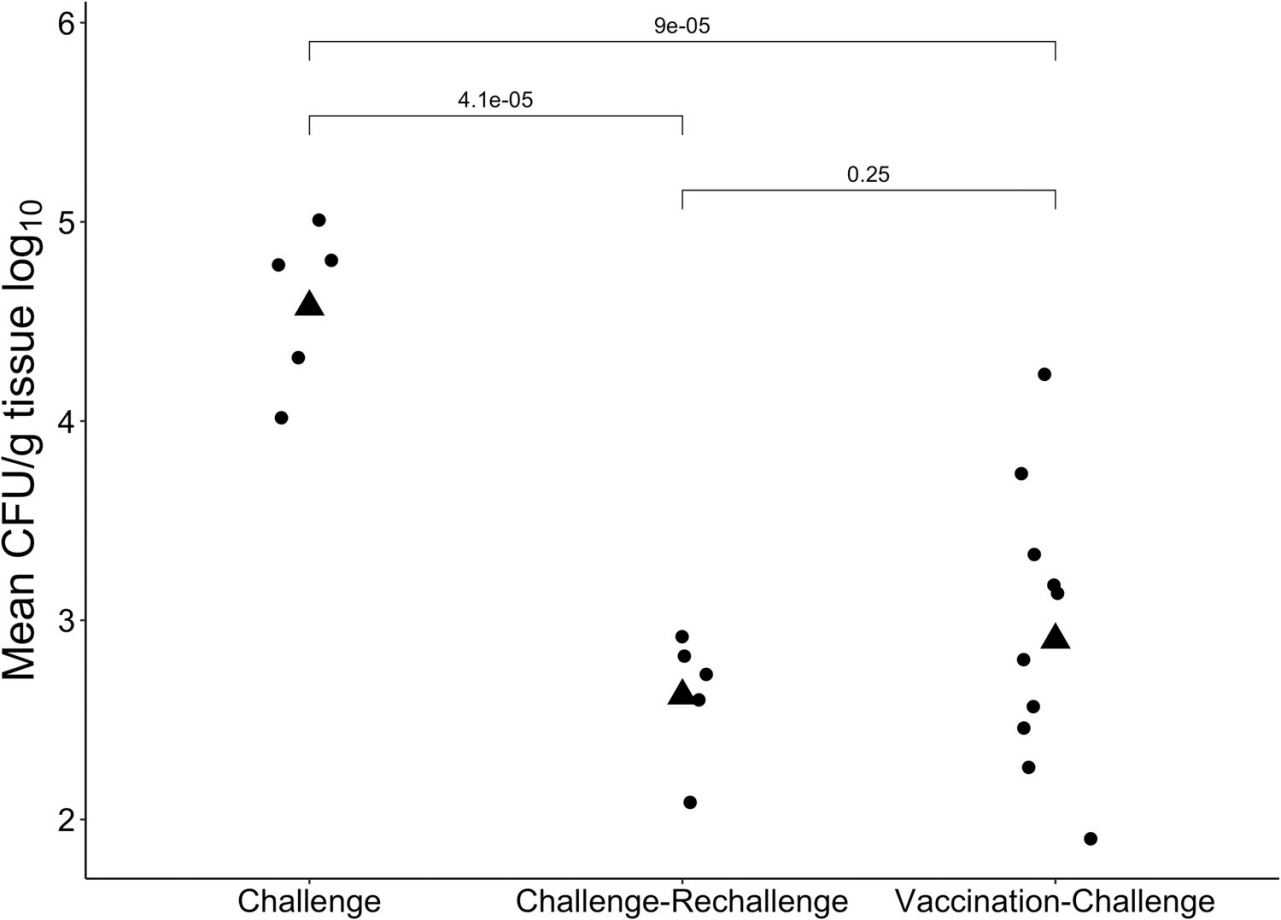
Mean CFU/g of ETEC strain B7A after naïve challenge, challenge followed by rechallenge, and vaccination followed by challenge. The challenge and rechallenge dose were 10^11^ CFU of strain B7A. Mean CFU/g for each rabbit is plotted as circles, with the geometric mean of the treatment group displayed as a triangle. P values shown are the result of Student’s T-tests comparing mean CFU/g of all rabbits in each treatment group.

Using strain B7A at a dose of 10^11^ CFU, we found that colonization of the distal small intestine was markedly lower if the animals had been previously challenged with this strain (Challenge-Rechallenge), or if they were immunized with three doses of the CFA/I/II/IV MEFA vaccine as described above (Vaccination-Challenge) (**Fig 3**). The geometric mean CFU values for each group are as follows: 1) naïve rabbits challenged with B7A - 385,675 CFU/g 2) rabbits previously challenged with B7A allowed to recover and re-challenged with the same strain– 4,304 CFU/g 3) vaccinated rabbits challenged with 10^11^ CFU of B7A – 9,079 CFU/g. The p-values when compared between groups are <0.05 when comparing naïve animals to either previously challenged animals or vaccinated animals, but there is no significant difference (p = 0.25) when comparing previously challenged and vaccinated animals. P values are the result of Student’s T-tests comparing mean CFU/g of all rabbits in each treatment group.

### Serum from vaccinated and challenged rabbits shows high IgG titers for ETEC adhesins and toxin

**Fig 4:**
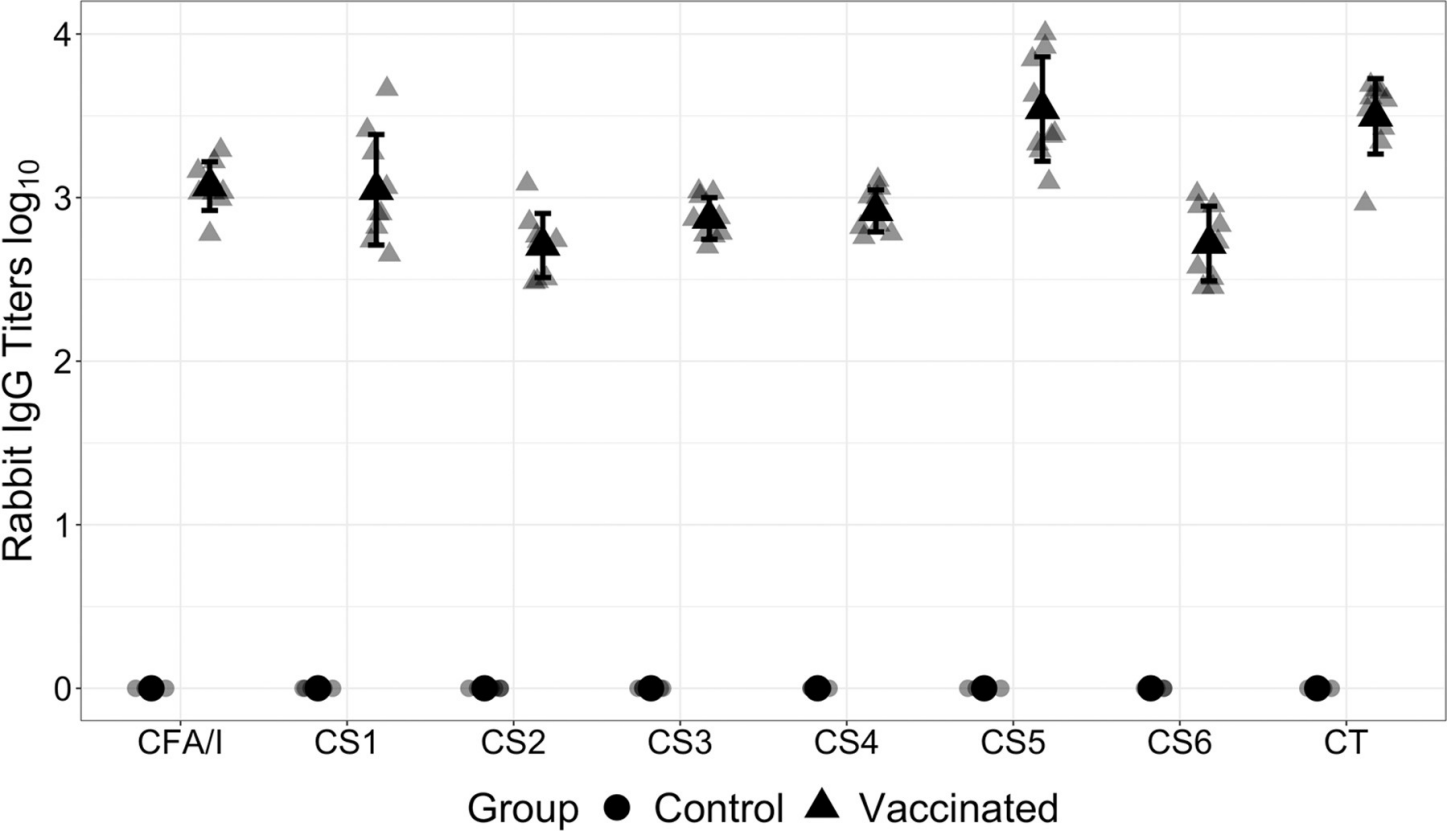
Rabbit serum IgG titers for control and vaccinated animals. Rabbit serum IgG titers (log_10_) to CFA/I, CS1, CS2, CS3, CS4, CS5, or CS6 adhesin and toxin CT (LTB) are shown in grey symbols. Serum samples of each rabbit IM vaccinated with CFA/I/II/IV MEFA protein, with dmLT adjuvant, were titrated in ELISAs with CFA/I, CS1, CS2, CS3, CS4, CS5 adhesin, CS6 CssA recombinant protein, or CT (for LTB) as coating antigens. Black symbols and bars in each group represent the means and standard deviations of IgG titers. Control serum is from non-vaccinated, non-challenged rabbits.

Serum from the Vaccination-Challenge group (**Fig 2**) of animals was analyzed for presence of IgG binding to each of the seven ETEC adhesins included in the MEFA protein, as well as LT toxin. Titers, when compared to control animals, were significantly higher in the immunized animals (**Fig 4**).

### Serum from vaccinated and B7A challenged rabbits prevents adherence of CS6-producing ETEC to Caco-2 intestinal epithelial cells

**Fig 5:**
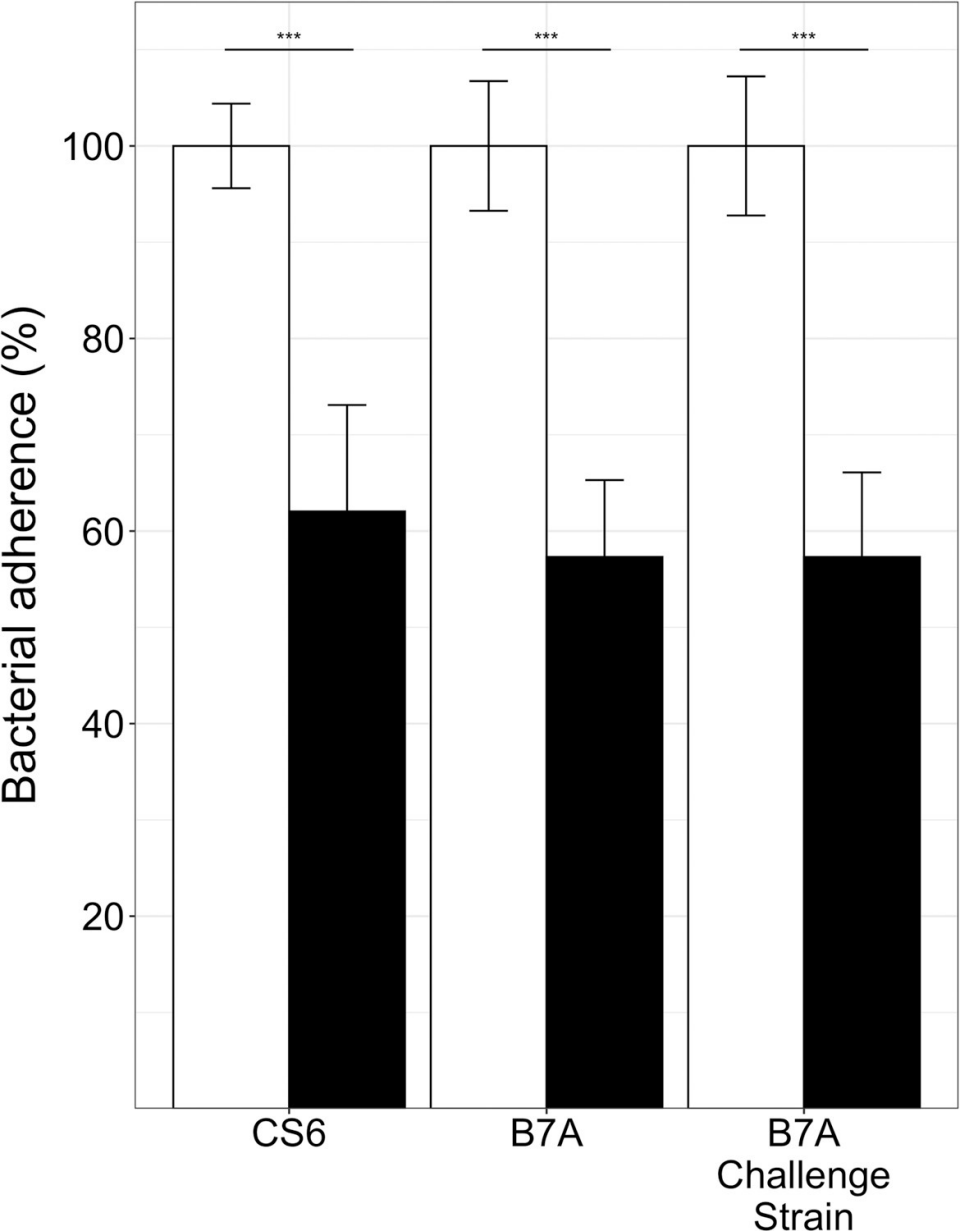
Rabbit serum antibody adherence inhibition activity against ETEC CS6 (2423 ETP98066) or B7A strains. CS6 and B7A bacteria adherent to Caco-2 cells (CFUs, in %) after incubation with rabbit serum samples from the control group (□) or the group IM vaccinated with CFA/I/II/IV MEFA protein (■). Boxes and bars represented means and standard deviations, *** indicates a p value of < 0.001.

Serum from the Vaccination-Challenge group (**Fig 2**) or control animals was used for a bacterial adherence assay using Caco-2 intestinal epithelial cells (**Fig 5**). The serum from vaccinated and challenged animals significantly inhibited the adherence of ETEC strains producing CS6 adhesin. These included two different stocks of strain B7A (a lab stock and GMP challenge stock), and another CS6+ ETEC strain: 2423 ETP98066 (**Table 1**).

## Discussion

Previous evaluations in other animal models suggested that the MEFA vaccinology platform to generating a multi-epitope vaccine on a single protein backbone for ETEC was promising [19]. Previous work in mice and pigs demonstrated that this CFA/I/II/IV MEFA antigen, combined with administration of a toxoid fusion (CFA/I/II/IV MEFA protein combined with toxoid fusion protein 3xSTa_N12S_-mnLT_R192G/L211A_ to constitute multivalent enterotoxigenic *E*. *coli* vaccine MecVax) was able to induce functional antibody responses in both of these model organisms [19, 37]. The results presented here add to the growing body of work in this arena, as we demonstrated the ability for the MEFA-based vaccine, in combination with dmLT adjuvant, to induce an immune response and to protect from B7A colonization of the rabbit small intestine.

The candidate vaccine adhesin antigen was well-received in New Zealand White Rabbits, rabbits were assessed daily, and we observed no adverse events when it was given intradermally or intramuscularly. In Fig 1, we showed a comparison of the titers in naïve animals that received a full vaccine dose given via either the intradermal (ID) or intramuscular (IM) route. We concluded that the response from these routes was not significantly different and chose to immunize IM for simplicity moving forward.

After establishing that the candidate vaccine antigen was safe and elicited a robust immune response in the rabbit model, we sought to establish an *in vivo* model for ETEC colonization using rabbits and infecting with well-characterized challenge strains (Table 1). We sampled the proximal and distal small intestine of infected, naïve animals to determine colonization levels. As can be seen in Fig 2, the bulk of the colonization in these rabbits was distal. Typically, ETEC is thought to colonize the proximal small intestine with the help of CFA adhesins [38]. Differences in our model may be attributed to significant structural and physiological differences in the gut of rabbits, specifically in O_2_ levels associated with ETEC virulence gene expression [39]. We also note the limitations of our sampling strategy, as the intestinal samples were vortexed rather than scraped or ground, the strength of attachment and the role this plays in the rabbit model is an area of ongoing investigation. Of the strains we included in our assay, the one with reliably high levels of colonization was B7A. We are currently planning follow-up experiments to determine if this is a CS6-dependent phenomenon and investigate methods of increasing the baseline colonization of heterologous strains such as buprenorphine treatment [40].

Using B7A as a challenge strain, we were able to demonstrate a significant reduction in colonization due to either homologous pre-challenge, or vaccination with the candidate MEFA/I/II/IV vaccine prior to challenge (Fig 3). A full course of vaccination in these animals was as effective as a previous exposure (Challenge-Rechallenge) at reducing the colonization of B7A. When we further investigated the serum of the vaccinated and challenged animals, we saw high titers of IgG antibodies against all seven of the adhesin components making up the MEFA/I/II/IV MEFA antigen, and anti-toxin antibodies (LT) (Fig 4). These antibodies were shown to be functional in that they prevented the adherence of ETEC strains to Caco-2 cells *in vitro* (Fig 5). The dmLT adjuvant used has been evaluated for its effect on immunogenicity in this context when used as a parenteral adjuvant with a MEFA/I/II/IV-based vaccine [41]. While anti-ST antibodies were not assayed directly in this study, they have been shown to be induced previously using a toxoid fusion-based approach in other model animals [21, 37].

Taken together, these results align well with other MEFA studies from mice and pigs. Compared with other vaccines, the flexibility of the MEFA approach allows for the inclusion of more heterologous CFA epitopes to be included, along with the possibility of including both LT and ST toxins in a single vaccination. The most encouraging data for use of an ETEC vaccine in the developing world have come from an oral inactivated whole-cell ETEC vaccine candidate (ETVAX), which combines four recombinant ETEC strains that overexpress CFAs [42]. However, with only four CFA adhesins represented in ETVAX, and no immunity against heat-stable enterotoxin (ST) which plays a more important role in children’s diarrhea and travelers’ diarrhea [4, 43, 44], as well as the relatively poor immune responses to oral vaccines for young children in developing countries, there are still challenges in the field that are left to be met [45].

In conclusion, we have demonstrated the use of a rabbit model for ETEC colonization. Using strain B7A, we have demonstrated that the candidate MEFA vaccine expressing epitopes for seven heterologous CFAs can protect against ETEC colonization in this model. There remains a need to evaluate this candidate vaccine for safety, immunogenicity, and protection in human volunteers. However, the potential of eliciting broad protection using a single protein is an exciting advancement for the field of vaccinology generally, and particularly for a problematic pathogen such as ETEC.

## Supporting information

S1 Data Elisa raw data. (XLSX)

S2 Data Colonization raw data. (XLSX)

S3 Data Inhibition raw data. (XLSX)
